# Conversion of lignocellulosic agave residues into liquid biofuels using an AFEX™-based biorefinery

**DOI:** 10.1186/s13068-017-0995-6

**Published:** 2018-01-16

**Authors:** Carlos A. Flores-Gómez, Eleazar M. Escamilla Silva, Cheng Zhong, Bruce E. Dale, Leonardo da Costa Sousa, Venkatesh Balan

**Affiliations:** 1 Departament of Chemical Engineering, Tecnológico Nacional de México, I. T. Celaya, Av. Tecnológico S/N, 38010 Celaya, Guanajuato Mexico; 2 Department of Chemical Engineering and Materials Science, Michigan State University, 3815 Technology Boulevard, Lansing, MI 48910 USA; 3 DOE Great Lakes Bioenergy Center, Michigan State University, East Lansing, MI 48823 USA; 4 Department of Engineering, Tecnológico Nacional de México, I. T. Roque, Km 8 Carretera Celaya-J. Rosas, 38110 Celaya, Guanajuato Mexico; 5 Key Lab of Industrial Fermentation Microbiology of Ministry of Education, School of Biotechnology, Tianjin University of Science & Technology, Tianjin, People’s Republic of China; 6 Biotechnology Division, Department of Engineering Technology, School of Technology, University of Houston, Houston, TX 77004 USA

**Keywords:** Agave, Biomass, Pretreatment, AFEX, Enzymatic hydrolysis, Cellulase, Fermentation, Biofuel, Lignocellulosic, Ethanol

## Abstract

**Background:**

Agave-based alcoholic beverage companies generate thousands of tons of solid residues per year in Mexico. These agave residues might be used for biofuel production due to their abundance and favorable sustainability characteristics. In this work, agave leaf and bagasse residues from species *Agave tequilana* and *Agave salmiana* were subjected to pretreatment using the ammonia fiber expansion (AFEX) process. The pretreatment conditions were optimized using a response surface design methodology. We also identified commercial enzyme mixtures that maximize sugar yields for AFEX-pretreated agave bagasse and leaf matter, at ~ 6% glucan (w/w) loading enzymatic hydrolysis. Finally, the pretreated agave hydrolysates (at a total solids loading of ~ 20%) were used for ethanol fermentation using the glucose- and xylose-consuming strain *Saccharomyces cerevisiae* 424A (LNH-ST), to determine ethanol yields at industrially relevant conditions.

**Results:**

Low-severity AFEX pretreatment conditions are required (100–120 °C) to enable efficient enzymatic deconstruction of the agave cell wall. These studies showed that AFEX-pretreated *A. tequilana* bagasse, *A. tequilana* leaf fiber, and *A. salmiana* bagasse gave ~ 85% sugar conversion during enzyme hydrolysis and over 90% metabolic yields of ethanol during fermentation without any washing step or nutrient supplementation. On the other hand, although lignocellulosic *A. salmiana* leaf gave high sugar conversions, the hydrolysate could not be fermented at high solids loadings, apparently due to the presence of natural inhibitory compounds.

**Conclusions:**

These results show that AFEX-pretreated agave residues can be effectively hydrolyzed at high solids loading using an optimized commercial enzyme cocktail (at 25 mg protein/g glucan) producing > 85% sugar conversions and over 40 g/L bioethanol titers. These results show that AFEX technology has considerable potential to convert lignocellulosic agave residues to bio-based fuels and chemicals in a biorefinery.

**Electronic supplementary material:**

The online version of this article (10.1186/s13068-017-0995-6) contains supplementary material, which is available to authorized users.

## Background

The development of lignocellulosic bio-based products including advanced biofuels is receiving increased attention in different parts of the world, due to sustainability and energy security merits [1, 2]. In addition, transforming locally available agro-industrial residues into liquid biofuels is key to reduce negative environmental impacts of fossil fuel consumption by the transportation section, promote regional economic development, and create rural employments [3–5].

Tequila and Mezcal are two Mexican spirit beverages, produced using fructan-rich agave stem juice. The beverage named tequila is only produced from *Agave tequilana weber*, whereas Mezcal is produced using diverse regional agaves species (*angustifolia*, *americana*, *salmiana*, among others). Both beverages are members of the *organization for an international Geographical Indications network* (“oriGIn”) and have international protection through appellation of origin (AO), recognized by NAFTA and the World Intellectual Property Organization (WIPO). Mezcal is exported mainly to the US and the EU, but Tequila has greater name recognition and is currently exported to more than 40 countries [6–8]. Although at present more than 90% of tequila production comes from the state of Jalisco, AO was given to five states in Mexico. However, currently Mezcal AO was increased to nine states of Mexico [7]. At present, the state of Oaxaca is the highest producer of Mezcal. However, other states in both AO regions are taking steps to increase the production of these spirit beverages.

**Fig. 1:**
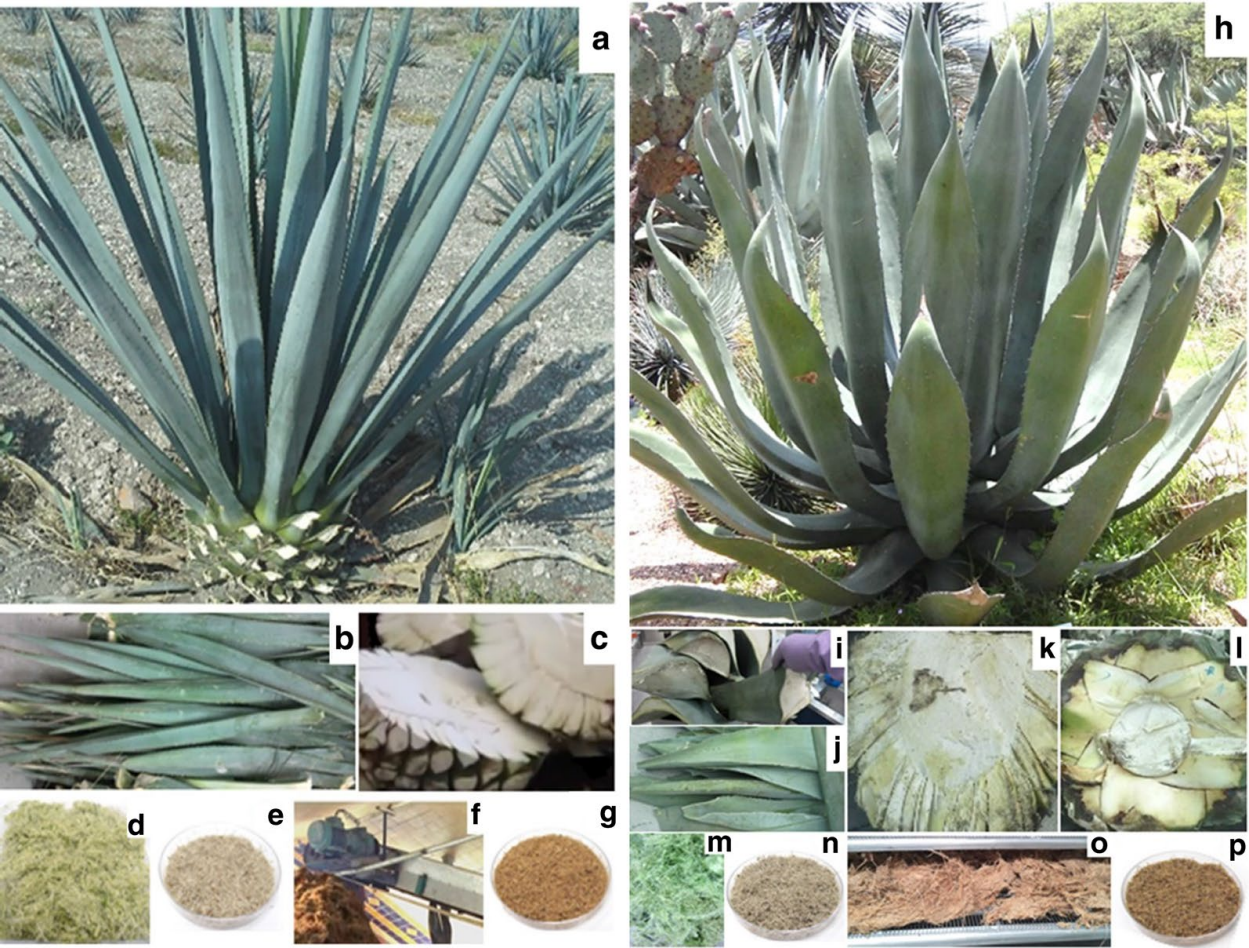
*Agave tequilana* and *Salmiana* plants and its main fractions. *A. tequilana* plant (**a**) and its harvested stem “piña” and leaves (**a**–**c**), leaf fiber matter (**d**, **e**), the stem-processed tequila residue “bagasse” (**f**, **g**). *A. salmiana* plant (**h**), *A. salmiana* leaves (**h**–**j**), stems (**k**, **l**), its leaf fiber matter (**m**, **n**), and the stem-processed Mezcal residue: *A. salmiana* “bagasse” (**o**, **p**)

Figure 1 provides details on two agave plants (*A. tequilana* and *Agave salmiana*), its main fractions (stem and leaves), and the lignocellulosic residues obtained from each (bottom). After harvest, the agave stems (called “piñas”) look like pineapples (Fig. 1c, k and l); however, *A. salmiana* stems are heavier than those of *A. tequilana* (> 2 times) and their succulent leaves are thicker than those from *A. tequilana*. The harvested agave stems are transported to the factories, where they are subjected to thermally assisted hydrolysis and juice extraction (mechanical and/or water-diffused). The fructose-rich juices are then fermented and distilled to beverage products, while the solid residues (known as bagasse) are left behind. In these residues, there are some left over sugars following juice extraction and might vary among processing facilities. Most of the bagasse generated by more than 800 agave-based alcoholic beverage factories is discarded as a solid residue [7, 9]. Only a small portion of bagasse is used for mulch, compost, or for heating applications. The agave leaves, also called “pencas” (Fig. 1a, b and h–j), are applied to their fields by the agave farmers because there is no current higher value use for these materials. Agave bagasse and leaf fiber represent more than 60% of the whole agave plant wet weight [10, 11]. These agro-industrial residues have considerable potential to produce bio-based fuels and chemicals in a biorefinery setting.

Since 2011, agave has received worldwide attention as a potential dedicated feedstock for producing liquid biofuels [12–15]. Agave species efficiently use water due to their specialized photosynthetic pathway known as crassulacean acid metabolism (CAM). CAM plants are adapted to grow under low water availability conditions. CAM plants can fix CO_2_ at night when the temperature is cooler and the relative humidity is higher than during the hotter day time, thereby reducing evapotranspiration losses [16]. Agaves are perennial evergreen xerophytes that can be cultivated in semi-arid lands where food/fodder crops cannot grow [15]. Growing perennial crops on these unproductive lands could improve the lives of rural populations who live in these dry areas [14]. Recently, Yan et al. [17] performed a life cycle assessment (LCA) study on agave-derived bioethanol. Their analysis suggests that agave-derived first-generation bioethanol is at least comparable with that from corn, switchgrass, and sugarcane in terms of energy and GHG balances, ethanol yield (95.3 L/Mg agave), and net GHG offset per unit land area.

Plant cell walls are naturally recalcitrant to invading pathogenic microbes and insect pests. There are two possible routes for producing biofuels from lignocellulosic feedstocks, namely the biochemical route and the thermochemical route [18]. The biochemical route requires a pretreatment step to disrupt the lignin–carbohydrate complex network, enable enzyme access, and hydrolyze polysaccharides into fermentable sugars [19]. Numerous pretreatment technologies have been developed in the past decades; however, few pretreatment processes allow high enough sugar conversions during enzyme hydrolysis to be considered viable [20]. Leading acidic pretreatments such as dilute acid, steam explosion, and liquid hot water provide promising sugar yields. However, these pretreatments are performed at elevated temperatures (> 160 °C), promoting the formation of sugar and lignin degradation products. These byproducts can inhibit enzymes and/or microbes [21–23] and negatively influence ethanol yields. To overcome inhibition, expensive detoxification steps are required prior to fermentation [22, 24–26]. Different methods, including washing, have been investigated to remove the inhibitory byproducts; however, the cost to remove inhibitors can be as high as 22% of the total ethanol production costs [26, 27].

Several pretreatments have also been reported for agave bagasse, including dilute acid, alkaline extrusion, and others [12, 28, 29]. However, low hydrolysis yields have been reported even at low solids loading conditions. In spite of high enzyme loadings, low ethanol concentrations were produced during fermentation. Recently, multi-step pretreatments have been used to raise sugar yields and fractionate biomass components (lignin, hemicellulose, and cellulose) into separate process streams, thereby reducing biomass recalcitrance toward enzymatic hydrolysis [30–32]. However, these pretreatments are expensive due to consumption of catalysts, solvents, energy, and water, and also generate waste streams, which are significant bottlenecks to economical bioconversion of agave residues.

In order to economically produce ethanol from lignocellulosic biomass, the following criteria should be met: (1) low energy and water usage during processing operations; (2) easy catalyst recycling during pretreatment, allowing easier downstream processing; (3) sugar conversions of > 85% at high solids loading (> 18%) without losing carbohydrates during the process; and (4) ethanol concentrations of at least 40 g/L to reduce the distillation costs. In particular, hydrolysis at high solids loading will reduce both capital and operating costs and increase the fermentable sugar concentration and the potential ethanol titers, thereby improving the economic viability of second-generation bioethanol production [33–36].

It is challenging to work with lignocellulosic biomass substrates at high substrate concentrations, since enzyme efficiency is reduced at elevated sugar concentrations. Poor mixing due to the nature of the substrate during enzyme hydrolysis and high slurry viscosities (which increase abruptly above 20% solids) are important aspects of the ‘high solids effect’ that substantially reduces sugar conversion [37, 38]. Other possible reasons for reduced sugar conversions during high solids enzyme hydrolysis include inherently poor mass transfer, reduced free-water availability for enzymatic action, and increased non-productive enzyme binding to lignin [33].

Ammonia fiber expansion (AFEX™)^1^ is a leading pretreatment that can increase sugar conversion in an effective and sustainable manner [18]. AFEX is a “dry-to-dry” alkaline treatment that requires minimum water inputs and does not generate liquid waste streams unlike most thermochemical pretreatments [39]. Feedstocks are exposed to liquid or gaseous ammonia in a pressure vessel at moderate temperatures, and after a short residence time, the pressure is released [40]. The catalyst (NH_3_) can be easily recycled (~ 97%) due to its high volatility [41, 42]. Other advantages of AFEX include the following: minimal loss of available sugars and limited formation of inhibitors compared to other pretreatments, no washing steps required, and preservation of inherent nutrients (proteins, vitamins, minerals, etc.) [43–45]. Furthermore, the residual ammonia adsorbed in the pretreated biomass can be utilized as a nitrogen source by downstream microbes, and thus the AFEX pretreatment provides highly fermentable lignocellulosic hydrolysates [40, 43].

AFEX has been proven effective on monocot grasses such as switchgrass, corn stover, and rice straw 44, 46]. Also, *Saccharomyces cerevisiae* 424A (LNH-ST) is an efficient metabolically engineered strain that ferments both glucose and xylose in AFEX-pretreated biomass hydrolysates at high ethanol yields [47, 48]. Agaves are succulent monocot plants and have 61.7% of its proteome in common with four other monocot grass species, including corn (*Zea mays*) [49]. Previous work showed that AFEX-pretreated agave bagasse produces the highest overall sugar yields during enzymatic hydrolysis, compared to ionic liquids and autohydrolysis pretreatments [50]. However, it is necessary to optimize AFEX process parameters in order to improve sugar and ethanol yields. Also, different parts of the agave plant and/or different agave species may require particular process parameters, due to the inherent heterogeneity of cell wall composition. On the other hand, diverse pretreatment technologies differ in how they modify the cell walls. Hence, optimizing the enzyme cocktail is also important to improve the economics of saccharification at high solids loading conditions for AFEX-pretreated agave residues.

In this work, we optimized ethanol production from agave residues (bagasse and solid leaf matter) using substrates from two agave species (*A. tequilana* and *A. salmiana*). To achieve this, we first analyzed the chemical compositions of bagasse and leaf biomass from both species. Then, we optimized AFEX pretreatment conditions for the four agave feedstocks using a response surface methodology. Following this step, we determined the optimal ratios of commercial enzymes (Cellic^®^ CTec3, HTec3 and Multifect^®^ Pectinase) for hydrolysis under high solids loadings (6% glucan, that is 17–20% total solids) using a statistical design of experiments approach. Finally, we evaluated the fermentability of each pretreated biomass hydrolysate using *S. cerevisiae* 424A (LNH-ST) strain.

## Results and discussion

### Composition analysis of agave bagasse and leaf matter

**Fig. 2:**
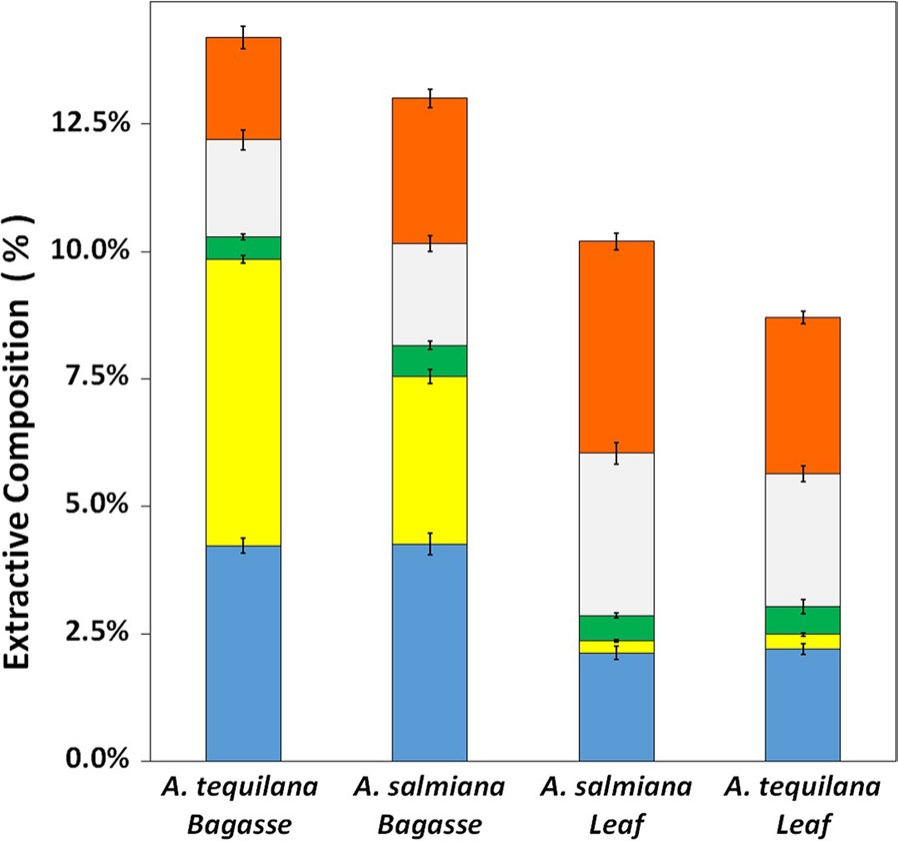
Composition of extractives on untreated agave lignocellulosic biomass. Water-soluble oligosaccharides (blue), water-soluble sugars (yellow), ethanol extractives (green), extracted protein (gray), and other extractives (red). Error bars represent the standard deviation of quintuplicates

The composition of water- and ethanol-soluble extractives of untreated agave lignocellulosic biomass is shown graphically in Fig. 2. The leaf fiber was found to have lower water-soluble carbohydrate (WSC) content than the bagasse. Also, *A. tequilana* bagasse contained more simple sugars, primarily fructose (data not shown). *A. salmiana* leaves had the highest content of ethanol extractives. Ethanol extractives of leaves contain chlorophyll and waxes [51], which came from the agave leaf surface. The total extractives (by ethanol and water) from *A. tequilana* leaf were lower than those from *A. salmiana* leaf (Fig. 2).

The composition analysis on extractives-free biomass is summarized in Table 1. The standard deviations reported here are from five replicates. High mass closures (> 97 wt%) for these samples were obtained by analyzing for total ash, total extractives, proteins, structural carbohydrates, acetyl residues, and Klason lignin. The glucan content for both agave species ranged between 30 and 35 wt% on a dry weight basis (DWB), consistent with previously published results [52]. In general, for both agave species, the xylan contents are slightly higher for bagasse than for leaf matter. However, *A. tequilana* bagasse had higher xylan content between the two species.

**Table 1:** Composition analysis of untreated agave biomass

Component	*Agave tequilana*		*Agave salmiana*	
	Bagasse	Leaf	Bagasse	Leaf
Ash	6.5 ± 0.3	7.5 ± 0.2	6.2 ± 0.5	7.6 ± 0.3
Extractives	14.2 ± 0.8	8.7 ± 0.4	13.0 ± 0.7	10.2 ± 0.4
Total protein^a^	3.8 ± 0.2	6.6 ± 0.4	4.4 ± 0.2	4.8 ± 0.4
Glucan	30.9 ± 0.7	35.0 ± 1.0	34.1 ± 1.0	35.2 ± 0.4
Xylan	13.0 ± 0.5	9.5 ± 0.5	12.0 ± 0.9	9.7 ± 0.5
Galactan	3.8 ± 0.5	3.9 ± 0.2	2.8 ± 0.1	4.0 ± 0.3
Arabinan	0.9 ± 0.2	2.1 ± 0.1	1.0 ± 0.1	2.4 ± 0.2
Mannan^a^	1.3 ± 0.2	2.9 ± 0.2	1.7 ± 0.2	2.0 ± 0.2
GA^a,b^	9.1 ± 0.6	9.6 ± 0.5	7.6 ± 0.8	9.7 ± 0.4
Acetyl	3.2 ± 0.4	2.3 ± 0.2	3.0 ± 0.3	2.2 ± 0.2
Klason lignin	12.9 ± 0.9	9.8 ± 0.6	13.0 ± 0.3	9.5 ± 0.5
Total	99.6	97.9	98.8	97.3

Results in percentage of DWB. Most data are reported as average of quintuplicates

^a^Average of triplicates

^b^GA as d-galacturonic acid homo-polymer

The composition analyses show significant levels of d-galacturonic acid, derived from galacturonan homopolymer (pectin) and ranging from 7.6 to 9.7%. This finding could explain the fact that a previously reported mass closure for agave was only around 85% [52]. The monosaccharide content in the acid-hydrolyzed samples was quantified using HPLC analysis to eluting on a Bio-Rad HPX-87 P column suitable for separation of pentoses and hexoses. Separation of xylose, galactose, and mannose is not possible using the HPX-87 H column [53]. This may explain the high xylan content in *A. tequilana* leaf fibers reported in a recent work [54]. Knowing the composition of the agave bagasse is extremely important for hydrolysis at high solids loadings, as high xylan and pectin contents sequester water in the solid phase, decreasing the availability of free water and potentially increasing the viscosity of the mixture [55]. The acid-insoluble lignin (Klason lignin) content was slightly higher for bagasse than for leaf fiber for both *A. tequilana* and *A. salmiana* species. We do not report acid-soluble lignin levels, due to concerns about the accuracy of the estimate, as no absorptivity has been reported for agave. When we scan the acid supernatant using a spectrophotometer, we obtain a maximum absorbance near 280 nm; however, this result could have been influenced by acid hydrolysis byproducts (i.e., HMF and furfural) or by other unknown components present in acid hydrolysates [56].

### AFEX pretreatment of agave lignocellulosic biomass

The AFEX pretreatment conditions evaluated here were chosen based on statistical design of experiments (DoE) to evaluate the effect of varying process parameters on agave residues’ deconstruction. The response variables were based on the percentage of sugar released during enzymatic hydrolysis (EH) using 15 mg total protein/g glucan of commercial enzymes cocktail containing cellulases and hemicellulases at a constant mass ratio and at low solids loading (~ 3% w/v). Pretreatment efficacy at low solids EH was evaluated to minimize the effect of sugar inhibition and high viscosity. Additional file 1: Figure S1 summarizes the release of monomeric sugars (both glucose and xylose) in varying AFEX pretreatment conditions under a DoE for each agave residue evaluated. The enzymatic hydrolysis of AFEX-pretreated agave leaf and bagasse was significantly higher than that of the respective untreated samples (lower triangles in Additional file 1: Figure S1). AFEX cleaves acetylated ester linkages, increases the porosity of biomass, and thereby increases enzyme accessibility to cellulose in biomass [50].

In general, sugar release after hydrolysis of AFEX-pretreated agave leaf fiber is slightly greater than that for AFEX-pretreated agave bagasse. It is widely accepted that lignin is a major contributor to cell wall recalcitrance [57], and therefore these differences can be explained by the fact that leaf fiber contains lower levels of Klason, lignin (Table 1). In addition, *A. salmiana* leaf gave slightly higher average sugar conversions relative to *A. tequilana* leaf fiber, and sugar conversions from bagasse were comparable for both agave species.

SEM images for untreated and AFEX-treated *A. tequilana* bagasse (see Additional file 1: Figure S2) reveal some physical changes due to pretreatment; in the untreated samples, we see some holes and cracks and these features increase by thermal processing of “piña” during tequila production. Compared to unpretreated bagasse fiber, the AFEX-treated bagasse fiber is swollen. We also observed thick deposits on the surfaces of the AFEX-treated biomass, probably due to ammonia-soluble material that was re-localized and deposited onto the biomass surface during pretreatment, following the evaporation of ammonia. The physical appearance of the AFEX-pretreated agave bagasse as shown in the micrographs is consistent with prior observations of AFEX-pretreated corn stover [58].

To evaluate the effect of AFEX pretreatment conditions on sugar conversion for *A. tequilana* bagasse (ATB), a Box–Behnken statistical design was performed by varying moisture (from 0.4 to 0.7 g H_2_O/g DM), temperature (from 100 to 140 °C), ammonia-to-biomass ratio (NH_3_/BM) (from 0.5 to 2 g NH_3_/g agave DM), and residence time (from 16 to 60 min). The central design point was conducted in sextuplicate for a total of 54 experiments, including 2 replicates for the peripheral points.

A response surface regression was performed on the experimental results from glucan and xylan conversions as a function of the pretreatment conditions. The regression coefficients considered for the second-order model (1) were selected by stepwise regression with an alpha-to-enter < 0.15, and are shown in Table 2. By analyzing the regression coefficients for percent glucan-to-glucose conversion (monomeric) from pretreated ATB, we found that temperature, NH_3_/BM, and moisture are the key factors that explain the variability of the experimental data. Reaction time had the smallest impact (*p* = 0.041) on glucose conversion. Interaction factors such as temperature against moisture content and NH_3_/BM against moisture also explain this variability. The final model adequately describes the data with an adjusted *R*^2^ value of 86%.

**Table 2:** Response surface analysis of AFEX pretreatment factors

Term	*Agave tequilana*				*Agave salmiana*			
	Bagasse^a,b^		Leaf fibers^c^		Bagasse^b^		Leaf fibers^c^	
	Coef.	*p*	Coef.	*p*	Coef.	*p*	Coef.	*p*
*β*_0_	− 161.7	0.000	90.8	0.000	− 108	0.000	74.1	0.000
*β*_1_	0.511	0.041	na		na		na	
*β*_2_	2.849	0.004	− 0.328	0.027	2.633	0.035	− 0.070	0.012
*β*_3_	11.10	0.000	17.79	0.000	19.01	0.000	22.24	0.000
*β*_4_	130.5	0.002	− 77.7	0.016	61.90	0.000	− 43.0	0.000
*β*_11_	− 0.0061	0.010	na		na		na	
*β*_22_	− 0.0114	0.000	na		− 0.0103	0.001	na	
*β*_33_	–	–	na		− 0.712	0.012	na	
*β*_23_	0.2404	0.002	–	–	–	–	− 0.097	0.010
*β*_24_	− 0.582	0.028	0.828	0.014	− 0.450	0.031	0.446	0.015
*β*_34_	− 59.50	0.000	− 11.15	0.000	− 14.77	0.000	− 15.22	0.000

Regression coefficients (*β*_*i*_): 0 = constant; 1 = time^a^ (min); 2 = temperature (°C); 3 = NH_3_ (g/g DM), 4 = H_2_O (g/g DM)

na, not applicable

^a^Time was a variable on *A. tequilana* bagasse only, and was held at 30 min on the other feedstocks

^b^Second order model (with quadratic terms) on both species bagasse, from Box–Behnken DoE

^c^First order model (linear) on both species leaves, from full-factorial DoE

**Fig. 3:**
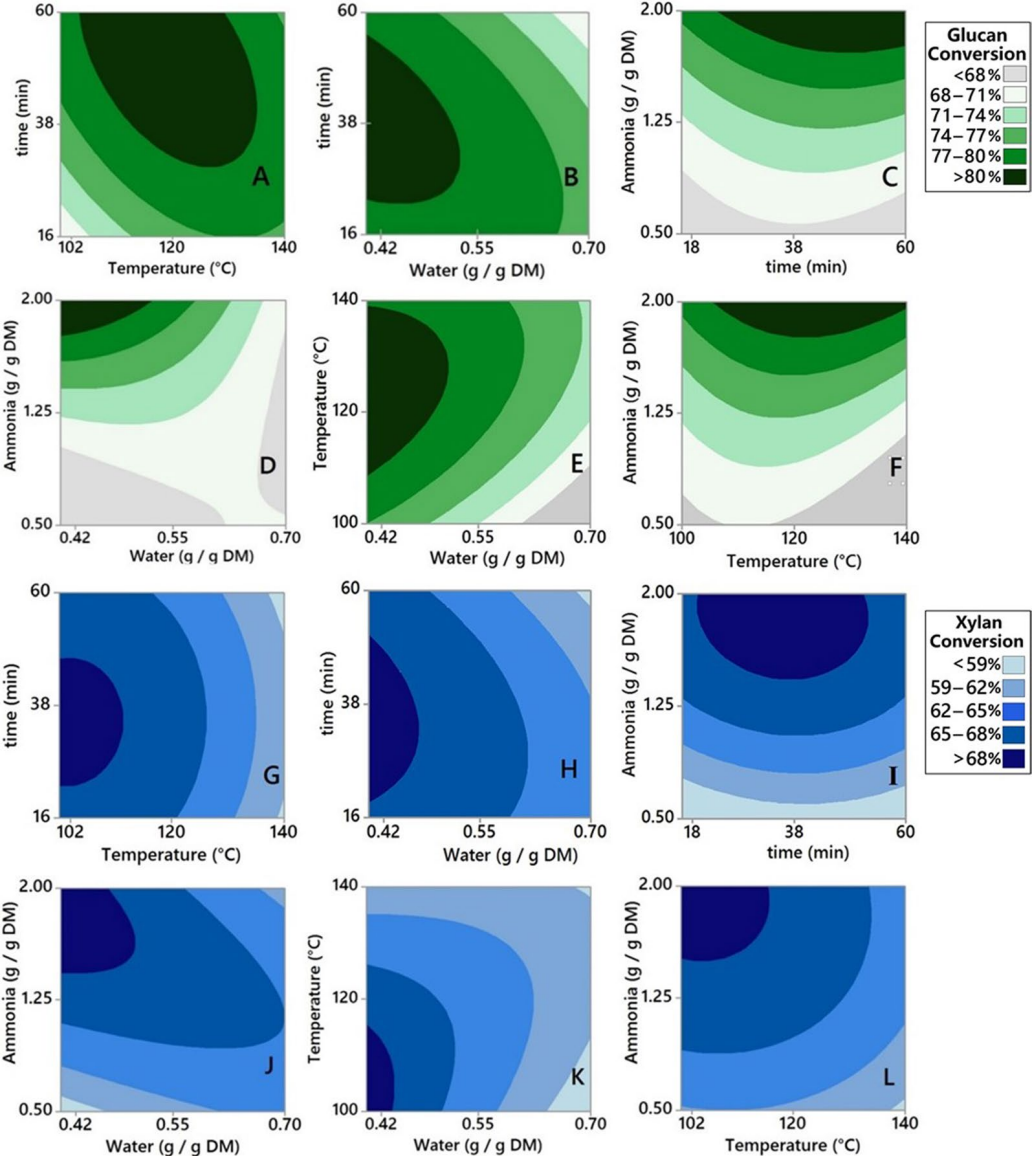
Effects of AFEX parameters on monomeric sugar conversions from *A. tequilana* bagasse. Contour plots showing the effects of varying pairs of pretreatment conditions on glucan-to-monomeric glucose conversion **A**–**F** (green) and xylan-to-monomeric xylose conversion **G**–**L** (blue) as a function of AFEX pretreatment parameters

Contour plots (Fig. 3) illustrate the effect of different AFEX conditions on glucan and xylan conversion from *A. tequilana* bagasse (ATB). While varying two AFEX parameters, the remaining two parameters were held constant at an optimal level from the range tested in our design of experiments. The dark areas indicate higher sugar conversions. Some AFEX conditions that gave higher glucose release (green contours) were roughly similar (overlap) to those for xylose release (blue contours). The glucose release for pretreated ATB as a function of temperature and time (Fig. 3A) was lower under lower severity conditions (lower left corner). It is widely believed that higher sugar conversions required more severe conditions, temperatures (> 120 °C), and long residence time (1–3 h). Higher temperatures usually favor the cleavage of lignin–carbohydrate complex (LCC) linkages, thereby promoting the necessary cell wall disruption [59–61]. However, higher temperatures during AFEX are known to produce lignin and sugar degradation products (inhibitors) [43] that negatively impact enzymes and microbes during hydrolysis and fermentation, respectively.

Thus, decreased sugar yields are observed for the higher temperatures tested here. From these experimental results, we found that intermediate severity AFEX conditions maximize sugar yields from ATB, thereby theoretically maximizing ethanol yield. It can be observed from Fig. 3D that the higher levels of ammonia-to-biomass ratio (NH_3_/BM) combined with low moisture contents give the highest monomeric glucan conversion within the boundaries of our experimental design. AFEX parameters for maximum glucan conversion were 0.4 g H_2_O/g DM moisture content (the lowest value tested), ammonia loadings > 1.75 g/g of DM, residence time of 38 min, and 120 °C pretreatment temperature.

Based on statistical modeling, higher carbohydrate conversions to monomeric sugars were obtained by keeping the reaction time (RT) around 30 min against the other factors tested (Fig. 3A, B, E, G, H, and K). From these analyses, we found that RT alone has a lower significance (*p* = 0.041) based on the range tested. In order to reduce the number of experiments, RT was kept constant at 30 min in subsequent experiments on the other agave residues tested (*A. tequilana* leaf fiber and *A. salmiana:* bagasse and leaf fiber). For *A. salmiana* bagasse (ASB), a 3-factor Box–Behnken DoE was used to evaluate the effect of varying temperature, moisture, and NH_3_/BM on glucan and xylan conversion, in the same ranges as for ATB (see Additional file 1: Table S1). A total of 30 experiments were run, including two replicates and 6 center points.

The regression coefficients of the model that predicts glucan and xylan conversions as a function of AFEX parameters on ASB were selected using the same methodology for ATB pretreatment experiments and are listed in Table 2. When these model terms were analyzed, we noticed that moisture content and NH_3_/BM both have an important influence on sugar conversion, as also does the interaction of these terms. Thus, the effect of NH_3_/BM on glucan conversion depends on the amount of moisture in the biomass, and this fact must be taken into account for agave residues’ pretreatment optimization. Likewise, the interaction between temperature and moisture content has statistical significance (*p* < 0.05).

Additional file 1: Figure S3 shows the effects of varying pretreatment parameters on sugar conversion for *A. salmiana* bagasse (ASB). In general, the same sugar conversion trends were observed for ASB (Additional file 1: Figure S3A–C) and ATB (Fig. 3D–F). For both agave bagasse species, the glucose release was greater when the ammonia-to-biomass ratio was higher and the moisture content of biomass was lower (Fig. 3D and Additional file 1: Figure S3A). These findings are consistent with the results reported by Sousa et al. [61] for corn stover: high ammonia loadings and low moisture content favor cellulose III (a highly reactive cellulose allomorph compared to native cellulose I) formation [61].

In light of these findings on agave bagasse pretreatment study (ATB and ASB), we proceed to vary the ranges of two other factors: biomass moisture (from 0.2 to 0.6 g H_2_O/g DM) and ammonia loading (from 1 to 3 g NH_3_/g DM), in order to optimize the AFEX conditions on *A. tequilana* leaf (ATL) and for *A. salmiana* leaf (ASL) fibers. We performed a 2^3^ full factorial DoE with two replicates, to estimate single component effects and their combinatorial interactions for each agave species leaf matter selected here. Pretreatment temperature was the same as in the agave bagasse experiments and RT was kept constant at 30 min. Four center points were included for a total of 20 experiments.

Each set of experimental results from AFEX pretreatment on ATL and ASL was fitted separately using first-order regression models. The factorial regression analysis results from both agave leaf matter samples are listed in Table 2. We found a strong interaction between ammonia loading and moisture content on both agave leaf fibers evaluated in this study. This interaction parameter seems to be a critical factor for sugar conversion on agave lignocellulosic biomass.

Contour plots were constructed for both ASL and ATL illustrating how pairs of pretreatment conditions affect both glucan and xylan conversion (see Additional file 1: Figure S4). We observed similar trends for sugar conversion as in the agave bagasse pretreatment experiments. However, we could achieve higher glucan conversions (~ 93%) combining 3 g NH_3_ and 0.2 g H_2_O/g of dry biomass at moderate temperatures on both agave leaf matters, using the same enzymatic hydrolysis conditions as in agave bagasse feedstocks. The improvement in sugar conversion could be due to lower lignin contents in agave leaves and the combination of high liquid ammonia-to-biomass ratio and low moisture content [61–63]. The optimal AFEX pretreatment conditions obtained for agave leaves and bagasse are listed in Table 3.

**Table 3:** Optimal AFEX pretreatment values obtained for agave lignocellulosic biomass

Agave biomass	Temperature (°C)	Ammonia (kg NH_3_/kg DM)	Water (kg NH_3_/kg DM)	Reaction time (min)
Tequilana bagasse	120	2	0.4	38
Salmiana bagasse^a^	102	2	0.4	–
Tequilana leaf^a^	100	3	0.2	–
Salmiana leaf^a^	100	3	0.2	–

^a^The reaction time was kept constant at 30 min, in the DoE

Although using higher ammonia-to-biomass ratio gave higher sugar conversion conditions, these are not necessarily the most economical conditions. Therefore, lower ammonia-to-biomass ratios for agave biomass were chosen for the next set of experiments (optimization of high solids loading enzymatic hydrolysis and fermentability tests). Using the regression models obtained in the pretreatment analysis (Eq. 1 and Table 2), the values used are 1.5, 2.0, 2.0, and 1.5 for *A. tequilana* bagasse, *A. tequilana* leaf matter, *A. salmiana* bagasse, and *A. salmiana* leaf matter, respectively.

#### Enzyme mixture optimization

The commercial enzyme cocktail Cellic^®^ CTec3 comprised cellulase enzymes expressed in *Trichoderma reesei* (including endo- and exo-cellobiohydrolases, accessory activities, bacterial beta-glucosidase, and minor amounts of hemicellulases) [64, 65]. Although Cellic^®^ CTec3 has some hemicellulases activity, we supplemented CTec3 with two other commercial enzymes, Cellic^®^ HTec3 (composed of xylanase and xylosidase activities as well as auxiliary enzyme activities) and Multifect^®^ Pectinase (composed of diverse hemicellulase activities such as arabinofuranosidase, xylan esterase, pectinase, pectin lyase, alpha galactosidase, mannanase, mannosidase, and other activities). We varied the enzyme combinations used to hydrolyze pretreated agave leaf and bagasse at high solids loading (6% glucan loading). Commercial enzyme combinations with fixed concentrations of total enzyme loading (20 mg total protein/g glucan) were evaluated using DoE.

**Fig. 4:**
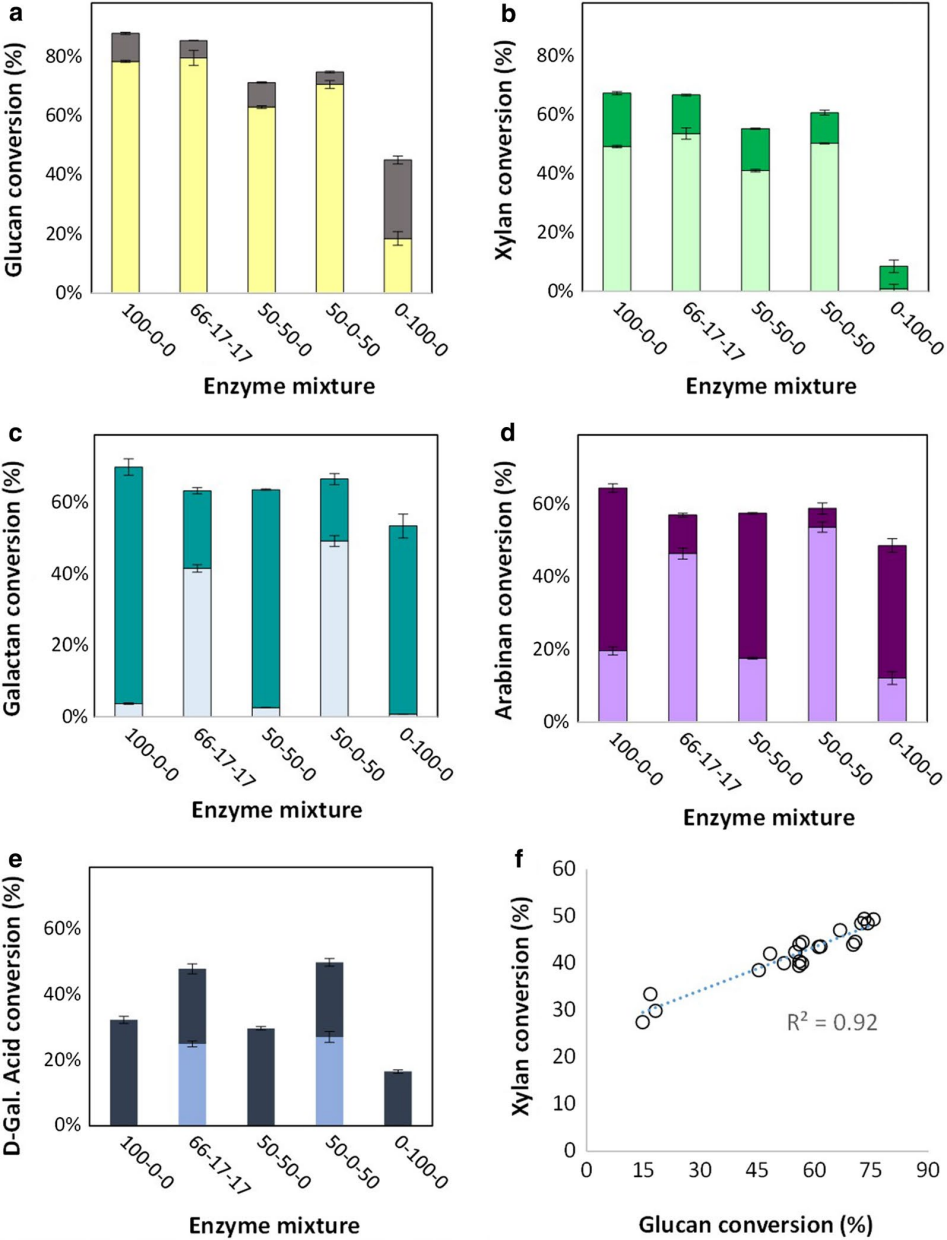
Effect of some enzyme ratio mixtures tested in EH of AFEX-pretreated *A. salmiana* leaf fiber. Bars represent the % of sugar conversion (monomeric in light and oligomeric in dark color bars): **a** glucose, **b** xylose, **c** galactose, **d** Arabinose, **e** d-galacturonic acid and **f** correlation between monomeric glucose and xylose release. EH at different enzyme ratios of CTec3–HTec3–multifect pectinase, at 20 mg total protein/g glucan (total enzyme loading), pH = 5.0, 50 °C, 250 rpm, and 72 h of reaction

Some enzyme combinations synergistically hydrolyze pretreated agave samples and gave higher sugar concentrations than others. Figure 4 shows the sugar conversions (monomeric and oligomeric) as a function of varying some enzyme ratio values under high solids loading conditions for enzyme hydrolysis of AFEX-pretreated *A. salmiana* leaf fiber. Only a few combinations produced more monomeric sugars as opposed to oligomeric sugars. Similar general trends for sugar conversions were observed on the other three pretreated agave biomass materials tested here (data not shown). Conversion to monomeric sugars is critical for ethanol production, as the yeast can only consume monomeric sugars. In Fig. 4f, we can see a strong linear correlation between the glucose and xylose released for each agave biomass. This strong correlation was noticed for *A. salmiana* bagasse and *A. tequilana* leaf and bagasse, with *R*^2^ values over 0.9 and *p* < 0.05 (data not shown). Similar correlations between glucose and xylose production were also observed for AFEX-pretreated corn stover and switchgrass [48, 66, 67]. These results suggest that glucan and xylan conversions are interdependent and that both cellulase and hemicellulase must be present in the cocktail to synergistically hydrolyze pretreated biomass. Compared to untreated agave biomass, ammonia helps chemically and physically modify the cell wall to enable greater accessibility of enzymes to the cell wall. Unlike dilute acid pretreatment, AFEX does not hydrolyze hemicellulose in the cell wall, and hemicellulase activities are crucial to enhance both glucan and xylan conversion [66]. Notably, galactose, arabinose, or d-galacturonic acid release did not correlate with either glucose or xylose release. When using Cellic^®^ CTec3 enzyme alone, we observed good monomeric glucose and xylose release, as CTec3 contains some hemicellulase activity [65]. However, the addition of small amounts of accessory enzymes to cellulase further enhanced the sugar conversion in pretreated agave biomass samples.

The coefficients of the mathematical model (Eq. 2, as stated in “Methods”) were estimated from regression analysis of the high solids loading enzymatic hydrolysis data for agave leaf fiber and bagasse (Additional file 1: Table S2). All terms have statistical significance (*p* < 0.05), including interaction terms. The sugar polymers hydrolyzed by the two accessory enzymes were predominantly non-cellulosic sugars. The effects of varying enzyme mixtures on monomeric sugar conversions from all four AFEX-treated agave biomass can be seen on the ternary contour plots at high solids loading (see Additional file 1: Figure S5).

However, since the glucose homopolymer (cellulose) is the main chemical constituent of agave leaf fiber and bagasse (Table 1) and based on the strong linear correlation observed between glucose and xylose release, we decided to use the optimal enzyme ratios obtained from the glucose release model for subsequent operations. Also, current ethanologens used to produce biofuels, including those strains engineered to metabolize xylose (*S. cerevisiae*, *Z. mobilis*, etc.), utilize glucose at a much higher rate than xylose, due to catabolic repression, the highly efficient glucose transporters in these organisms, and the redox imbalance [68, 69]. Therefore, the enzyme mixture used for subsequent experiments was set at 80:20 of CTec3 and HTec3. At the moment, the commercial enzyme cocktail Multifect^®^ Pectinase is no longer available at DuPont Corporation.

#### Effect of enzyme loading on HS enzymatic hydrolysis of pretreated agave biomass

The effect of enzyme loading on the sugar conversion for pretreated *A. tequilana* leaf fiber at 20% solids loading was studied. The CTec3-to-HTec3 ratio was kept constant and only the enzyme loading (10–30 mg protein/g of glucan) was varied. From the results (see Additional file 1: Figure S6), it is clear that beyond adding 25 mg protein loading/g of glucan of enzymes, there was no further improvement in sugar conversion. Similar behavior was observed in *A. salmiana* leaf and bagasse from both species (data not shown). Therefore, in subsequent experiments on separate hydrolysis and fermentation (SHF) of AFEX-pretreated agave residues 25 mg total protein/g of glucan was used. Although the amount of enzyme loading used in our experiments is higher, identifying the right combination of accessory enzymes in the future will further reduce the enzyme loading.

#### Fermentability test on AFEX-pretreated biomass at high solids loadings

**Fig. 5:**
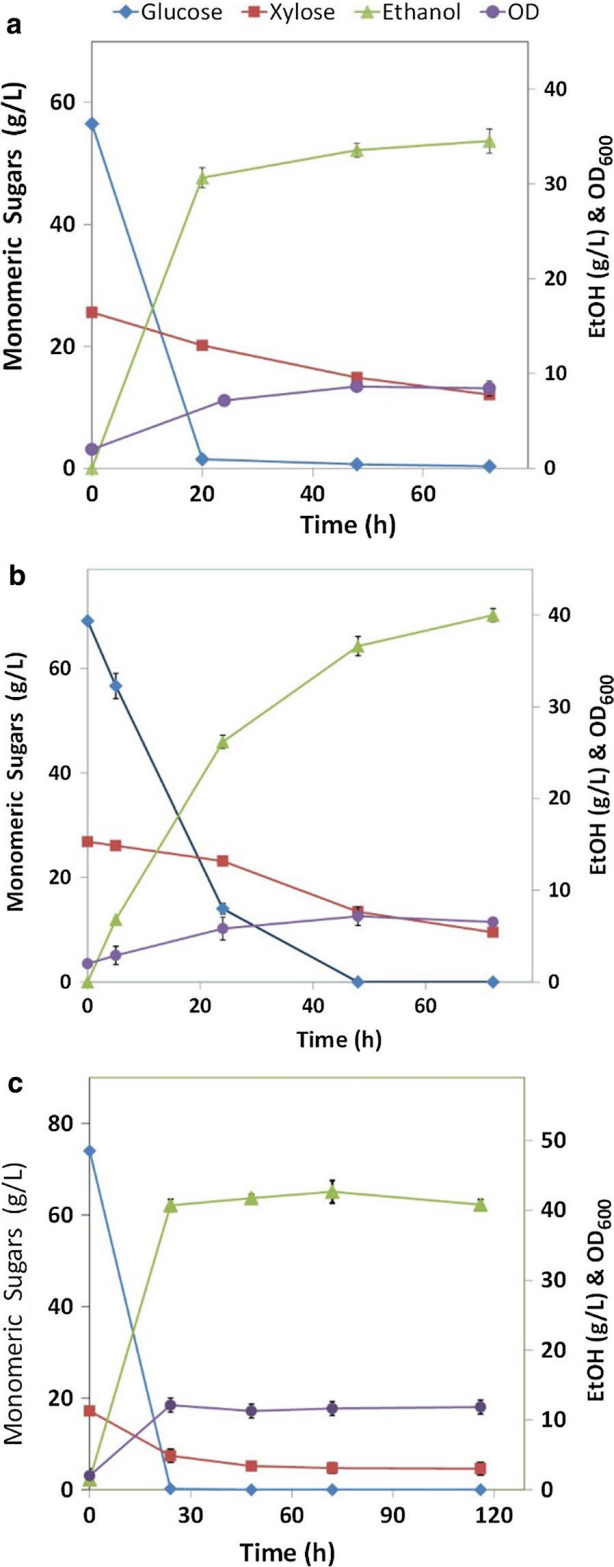
SHF performance with *S. cerevisiae* 424A on AFEX-treated agave biomass. Enzyme hydrolysis at ~ 20% total solids of AFEX-treated agave biomass. **a** *A. tequilana* bagasse, **b** *A. salmiana* bagasse, and **c** *A. tequilana* leaf matter

Separate hydrolysis and fermentations were conducted using AFEX-pretreated *A. tequilana* and *A. salmiana* (bagasse and leaf matter) using a 20% solids loading hydrolysate (Fig. 5), without using any washing, conditioning, or detoxification steps following pretreatment. Nor were any external nutrients added to the hydrolysate to assist the fermentation. Clearly, *A. tequilana* (bagasse and leaf fiber) and *A. salmiana* bagasse showed better sugar-to-ethanol conversion with higher metabolic rates than did *A. salmiana* leaf matter. Nutrients present in pretreated agave leaf fiber hydrolysates are sufficient to support yeast growth and ethanol production during fermentation in spite of the fact that the hydrolysate contains lignin degradation products. It is known that AFEX produces relatively low levels of degradation compounds compared to dilute acid pretreatment, although acetamide is one of the degradation products that is generated at higher concentrations when ammonia reacts with acetyl ester linkages [43].

We found that *A. salmiana* leaf hydrolysates could not be fermented to ethanol by *S. cerevisiae* 424A. One possibility is the presence of saponins, natural inhibitors present in the leaf fiber of many plant species. These saponins are amphipathic glycosides grouped phenomenologically by the soap-like foaming they produce when mixed in aqueous solutions. They have one or more hydrophilic glycoside moieties combined with a lipophilic triterpene derivative. Saponins are produced in plants to defend against invading microbes such as fungi [70]. Villegas-Silva et al. [71] report that *S. cerevisiae* did not grow in the enzyme hydrolysates of non-structural carbohydrates (fructans) from *Agave fourcroydes* leaf juice. In Mezcal production, only agave stems are processed with scant leaf matter included. Thermal industrial processing of the stems reduces the saponin content (saponins are hydrolyzed to a sapogenin and sugar), and water extractives formed during the first steam “cooking” process are discarded [72]. Because the *A. salmiana* bagasse underwent thermal processing at Mezcal factories, it presumably does not contain saponins. We did not measure saponin hydrolysis during AFEX pretreatment and more study is needed to understand the nature of the inhibitors in *A. salmiana* leaf hydrolysates. Some fungi have apparently been developed that produce detoxifying enzymes [71], and *S. cerevisiae* might also potentially be engineered to resist saponins [73].

### Mass balance during enzyme hydrolysis and microbial fermentation

**Fig. 6:**
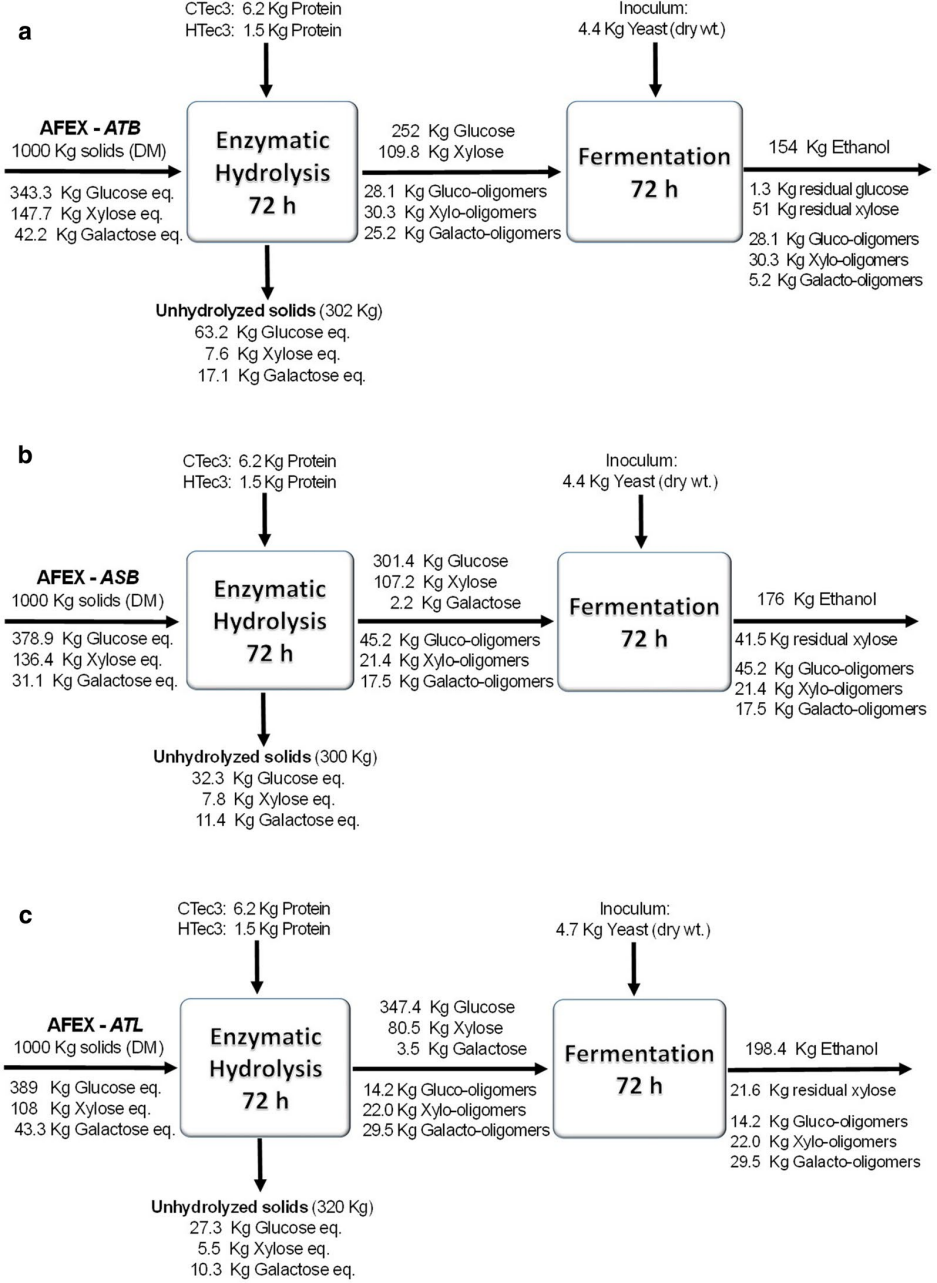
Mass balance for the EH and SHF on AFEX-pretreated agave residues: **a** *A. tequilana* bagasse, **b** *A. salmiana* bagasse, and **c** *A. tequilana* leaf fiber. Data were collected from ~ 20% w/w total solids

We have performed mass balance for converting agave residues into ethanol (Fig. 6). Since AFEX is a dry-to-dry process, we do not lose any biomass components into a separate liquid stream. In other words, there was complete recovery of solids after AFEX pretreatment. When 1000 dry kg of AFEX-treated *A. tequilana* leaves are subjected to enzyme hydrolysis, about 347 kg of glucose and 80 kg of xylose are produced (Fig. 6c). After 72 h hydrolysis, about 320 kg of unhydrolyzed solids (UHS) remain. The sugar polymers present in the UHS are highly recalcitrant and could not be converted to fermentable sugars. However, UHS could be used as a feedstock for a variety of biorefinery applications and/or electricity co-generation due to its better heating values (high remaining lignin content). About 4.4 kg of dry yeast was used to ferment the hydrolysate. About 22 and 30 kg of gluco- and xylo-oligomers, respectively, present in the hydrolysate are not utilized by the yeast and remain in the fermentation broth. In addition to oligosaccharides, about 21.6 kg of xylose remains in the fermentation broth for every 1000 kg of dry biomass input. More work is required to understand why such high concentrations of oligomeric sugars remain following enzyme hydrolysis and complete xylose consumption is not possible. Overcoming these two bottlenecks will further increase the ethanol yield. Figure 6a, b show mass balances for the *A. tequilana* and *A. salmiana* bagasse process. Overall, the amounts of ethanol produced from 1000 kg of different agave AFEX-treated substrates are given in brackets, *A. tequilana* bagasse (154 kg), *A. salmiana* bagasse (176 kg), and *A. tequilana* leaf fiber (198.4 kg).

As reported before, inhibitors that are produced during AFEX treatment affect enzyme hydrolysis due to non-productive enzyme binding and thereby lower sugar and ethanol yield. Also, the Maillard reaction products (between WSC and ammonia) could affect the yeast growth [43]; although in this work we did not examine Maillard byproducts, its presence is evident in bagasse and leaf matter (darker brown in AFEX-treated) samples as shown in Additional file 1: Figure S7.

## Conclusions

Bagasse and leaf fiber of two different agave species (*A. tequilana* and *A. salmiana*) were evaluated for ethanol production after pretreatment by AFEX, enzymatic hydrolysis, and microbial fermentation. Leaf fiber from both agave species contained lower lignin levels and were found to be highly digestible after AFEX pretreatment compared to their respective AFEX-treated bagasse samples. The optimal pretreatment conditions to enable high yields of fermentable sugars obtained from RSM were 0.4, 2.0, 120, and 38 for *A. tequilana* bagasse, 0.4, 2.0, 102, and 30 for *A. salmiana* bagasse, and 0.2, 2.0, 100, and 30 for both *A. tequilana* and *A. salmiana* leaf matter (g H_2_O/g DM, g NH_3_/g DM, °C, min), respectively.

Regarding high solids loading enzymatic hydrolysis optimization, the optimal commercial enzyme ratios DoE (CTec3:HTec3: multifect pectinase) from mixture, according to the proposed Minitab^®^ model. For 6% glucan loading (17–19% TS), of *A. tequilana* bagasse were 78:22:0, for *A. salmiana* bagasse they were 67:27:6; for *A. tequilana* leaf they were 80:18:2 and for *A. salmiana* leaf they were 78:10:12, the total enzyme loading was held constant at 20 mg protein/g glucan. On the other hand, the pretreated agave residues were fermented by SHF process using *S. cerevisiae* 424A strain, the total solids loading was increased to 20%, and the enzyme ratio was kept constant at 80:20 (CTec3:HTec3) at 25 mg total protein/g glucan. Except for *A. salmiana* leaf material, all other agave hydrolysates could be fermented to ethanol with high metabolic yields based on the mass balance (Fig. 6). The metabolic yields (calculated as g EtOH/g sugar metabolized × 0.51) were 97, 93, and 94% for *A. tequilana* bagasse, *A. salmiana* bagasse, and *A. salmiana* leaf, respectively. The amounts of ethanol produced from 1000 kg of different agave AFEX-treated substrates are given in brackets, *A. tequilana* bagasse (154 kg), *A. salmiana* bagasse (176 kg), and *A. tequilana* leaf fiber (198.4 kg). However, considerable amounts of gluco- and xylo-oligomers remain after enzyme hydrolysis, probably because the enzyme cocktail lacks key enzymes. Also, some xylose was left behind after 48-h fermentation for the bagasse samples. Additional research is required to understand this phenomenon and to overcome this bottleneck.

From this study, it is clear that the pretreatment technology impacts all other unit operations in a biofuel process. We have demonstrated that by using AFEX-based biorefinery it is possible produce bioethanol in Mexico from low-value agave bagasse and leaf matter. Following pretreatment, with the help of enzyme hydrolysis and microbial fermentation we could achieve sugar conversions > 85% at high solids loading (> 18%) without losing carbohydrates as waste stream and achieved ethanol concentrations of ~ 40 g/L. This is very important to economically produce ethanol from lignocellulosic biomass. Also, implementing such biorefinery operations will reduce the dependence on fossil fuel and reduce greenhouse gas emissions, increase energy security, benefit the environment, and promote regional and local economic development.

## Methods

### Feedstocks and conditioning

All the lignocellulosic biomass used in this study came from the state of Guanajuato in Mexico. *A. tequilana* bagasse (TB) was obtained from Tequila Corralejo factory, and *A. tequilana* leaf fiber was obtained from the “Universidad de Guanajuato.” *A. salmiana* bagasse was obtained from Zauco Mezcal factory, and *A. salmiana* leaf fiber was a generous gift from an agave farmer in the same area. These are representative samples and the composition of these biomass could change like any other species depending on the place, season they were harvested, and time of harvest. Agave leaf fibers of both species were further processed by passing through a shredder. The processed juice can be used for other application. However, in this study, we simply discarded the juice and did not use in any of the experiments. In this paper, the term “bagasse” refers to the lignocellulosic industrially processed agave stem (“piña”) from the two agave species. All the lignocellulosic materials were washed with deionized warm water in a ratio of 1:10, simulating the industrial processing conditions. The wet agave leaf fiber was squeezed (repeated at least 3 times) using a press, in order to remove most of the water-soluble sugars. The washed biomass was dried at 40 °C in a tray dryer until a moisture content < 10% was reached. This dry fibrous material was further milled in a cutter mill, sieved to obtain particle sizes ranging from 0.5 to 2 cm (length), and stored at 2 °C until use.

### Composition analysis of agave lignocellulosic biomass

Composition analysis was performed for lignocellulosic agave residues using National Renewable Energy Laboratory (NREL) protocols. The particle size was reduced in a centrifugal mill (Model ZM 200, Retsch, Newtown, PA) with a 2 mm screen, and the fraction retained on the 80 mesh sieve (−20/+80 mesh fraction) was used for compositional analysis [74]. Agave biomass was extracted using water, followed by ethanol (95%), to remove soluble non-structural materials at high pressure [51] using an Accelerated Solvent Extractor (ASE 200 Dionex, USA). Monomeric and oligomeric sugars were determined in the water fraction by HPLC using the protocol given below. Structural polysaccharides and acid-insoluble lignin present in the dry extractives-free biomass were determined by the standard two-step acid hydrolysis procedure [75].

### Protein content

Crude protein was calculated based on nitrogen content in the biomass, measured using a Skalar Primacs SN Total Nitrogen Analyzer (Breda, The Netherlands) and multiplying the nitrogen value by a factor of 6.25 [76].

### HPLC analysis

All sugars were analyzed using an HPLC system consisting of a Shimadzu LC-2010 (Milford, MA) equipped with a Waters 410 refractive index detector. Two columns were used to determine the composition of agave samples. The concentrations of different sugars such as sucrose, glucose, xylose, arabinose, mannose, and fructose were analyzed using a Bio-Rad Aminex HPX-87P column (Hercules, CA, USA) with a de-ashing guard cartridge (Bio-Rad Sunnyvale, CA, USA). Degassed HPLC grade water was used as the mobile phase at 0.6 mL/min at a column temperature of 85 °C. d-Galacturonic acid and acetate were analyzed during composition analysis using an Aminex HPX-87H column (Bio-Rad, USA). The same column was used to analyze fermentation broth including ethanol, xylitol, lactate, glucose, and xylose. A 5 mM aqueous sulfuric acid solution was used as the mobile phase at 0.6 mL/min at a column temperature of 60 °C. Xylose, galactose, and mannose peaks cannot be effectively separated using the HPX-87H column [77], and thus the results reported for xylose in fermentation broth may include mannose and galactose.

### Scanning electron microscopy (SEM)

SEM imaging of untreated and AFEX-treated *A. tequilana* bagasse was performed using a JSM-6060LV JEOL microscope at an accelerating voltage of 30 kV. Samples were mounted on metal stubs and were vacuum-coated with a thin layer of gold using an EMS 550 sputter coater.

### AFEX pretreatment design of experiments (DoE) and statistical analysis

Statistical experimental designs using Minitab Statistical Software (Minitab Inc., USA) were employed in this work to evaluate the effect of AFEX pretreatment variables on the sugar release from two agave species (*A. tequilana* and *A. salmiana*) and two parts of the agave plant (bagasse and leaf fiber).

For both agave bagasse species, Box–Behnken experimental designs were conducted and the following independent variables were analyzed: catalyst concentration (g NH_3_/g dry matter), moisture content (g H_2_O/g dry matter), temperature (°C), and residence time (min), with two replicates. Glucan and xylan conversions (g sugar released per gram of theoretical sugar in the dry biomass) were used as the responses. For *A. tequilana* bagasse, a total of 54 experiments (4 factors) involving 6 central points were performed. For *A. salmiana* bagasse, the reaction time was held constant at 30 min and 3 factors were varied; thus, a total of 30 experiments involving 6 central points were done. The value range for every independent variable is listed in Additional file 1: Table S1.

To obtain the mathematical models that predict glucan and xylan conversions (into monomeric glucose or xylose) after enzymatic hydrolysis of pretreated agave bagasse, experimental data points were subjected to surface response analysis using the following full quadratic equation:

1 $$ y = \beta_{0} + \mathop \sum \limits_{i = 1}^{n} \beta_{i} x_{i} + \mathop \sum \limits_{i = 1}^{n} \beta_{ii} x_{i}^{2} + \mathop \sum \limits_{i = 1}^{n} \mathop \sum \limits_{{\begin{array}{*{20}c} {j = 1} \\ {j \ne i} \\ \end{array} }}^{n} \beta_{ij} x_{i} x_{j} + \varepsilon . $$

Here, *y* is the response (% sugar conversion), *β*_0_ is the regression constant, *β*_i_ is the *i*th linear regression coefficient, *β*_*ii*_ is the quadratic regression coefficient, β_*ij*_ is the interaction coefficient for the *i*th and *j*th components, *x*_*i*_ and *x*_*j*_ are the *i*th and *j*th independent variables, respectively, *n* is the number of factors, and *ε* is the experimental error.

For leaf fibers from both agave species, two-level full factorial designs (2^3^) with two replicates and four center points were used to estimate single component effects and their combinatorial interactions relative to glucan and xylan conversion as a function of AFEX treatment conditions. The models for sugar conversion as a function of AFEX conditions were obtained from factorial regression analysis using a first-order polynomial equation. The statistical significance of the model terms is considered when the *p* value is < 0.05. Contour plots were generated based on the respective models, showing the effect of pairs of pretreatment parameters on sugar yields. The regression models were used to determine the optimum pretreatment conditions. AFEX conditions were considered optimal when the highest glucose plus xylose (monomeric) yields were obtained after EH.

### AFEX pretreatment optimization

Pretreatment experiments were carried out in 22 mL stainless steel reactors as previously described [78]. Briefly, lignocellulosic agave residues were sprayed with the desired amount of water and mixed. About 3 g DM of biomass was loaded into the reactor and sealed, and then the reactor was coupled to a vacuum pump for air removal. Anhydrous liquid ammonia was loaded using a syringe pump (Harvard apparatus-model PHD 2000, USA), and the reactors were heated immediately using a heating block, until the desired temperature was reached. After keeping the reactor at target temperature for a given residence time, the pressure was abruptly released. Finally, the pretreated biomass was transferred to aluminum trays and kept in a fume hood overnight.

To evaluate the AFEX effectiveness under different conditions, the pretreated agave samples were enzymatically hydrolyzed in 20 mL screw-cap vials and 15 mL total volume, and pH was adjusted to 4.8 using 1 M citrate buffer solution with sodium azide (10 mM) to prevent fungal and bacterial contamination. The enzyme cocktail consisted of Cellic^®^ CTec2 and HTec2 (Novozymes) at 9 and 6 mg protein/g glucan, respectively. Hydrolysis was performed for 72 h at 50 °C and 250 rpm.

### AFEX pretreatment for enzyme mixture optimization and fermentability test

Appropriate AFEX conditions were used to pretreat *A. tequilana* and *A. salmiana* bagasse and leaf fibers in a 3.9 L Parr reactor as described by Balan et al. [40]. AFEX-pretreated agave biomass was used in the study without any washing, detoxification, conditioning, or nutrient supplementation. It has to be noted that in addition to residual ammonia present in pretreated biomass, sugars present in CTec3 and HTec3 as storage stabilizers [64] could serve as yeast growth supplements.

### Design of experiments for enzyme hydrolysis optimization

To evaluate the effect of three commercial enzymes cocktail on monomeric sugar release throughout EH at high solids loading, a mixture DoE (augmented simplex-centroid) was created for all four feedstocks (pretreated leaf fibers and bagasse both for *A. tequilana* and *A. salmiana*) using Minitab. The responses were glucan and xylan conversion. The following polynomial equation was fitted for each of the four different agave biomass materials:

2 $$ y = \mathop \sum \limits_{i = 1}^{n} \beta_{i} x_{i} + \mathop \sum \limits_{i = 1}^{n}  \mathop \sum \limits_{{\begin{array}{*{20}c} {j = 1} \\ {j \ne i} \\ \end{array} }}^{n} \beta_{ij} x_{i} x_{j} + \varepsilon . $$

Here, *y* is the sugar yield, *β*_*i*_ is the linear regression coefficient for the *i*th component, *β*_*ij*_ is the quadratic interaction coefficient for the *i*th and *j*th components, *x*_*i*_ and *x*_*j*_ are the values of the *i*th and *j*th components, respectively, and *n* is the number of components (3 in this study). The terms considered for the regression model are those with *p* < 0.1. The final model was then used to understand the enzyme ratio optimization results and to predict the sugar yields for each pretreated agave biomass as a function of the proportions of all three enzymes. Contour plots were generated to observe the effect of commercial enzyme cocktail combinations on monomeric sugar yields. Although there are more sugars released than just glucose and xylose, the model was optimized for the highest release of those two monomeric sugars only.

### Enzyme mixture optimization

For optimizing ternary commercial enzyme mixture experiments at 6% glucan (17–20% of total solids loading), the AFEX-pretreated agave samples were hydrolyzed in 125 mL Erlenmeyer flasks with a total reaction mixture of 25 g. All samples were adjusted to pH 5.0, using 1 M citrate buffer solution with sodium azide (10 mM) to prevent contamination during the reaction. Cocktails with Cellic^®^ CTec3 and HTec3 (Novozymes) and Multifect^®^ Pectinase (Genencor) were mixed in different ratios as designed by the mixture DoE. The total enzyme loading was held constant at 20 mg of protein/g glucan. The hydrolysis conditions were 50 °C, pH 4.8, 250 rpm, and 72 h.

### Enzymes’ concentrations

The crude protein concentration for the enzymes was determined using the Kjeldahl nitrogen analysis method (AOAC) and the concentration of each commercial enzyme is given in brackets, CTec3 (210.6 g/L), HTec3 (164.6 g/L) and Multifect Pectinase (72 g/L). The densities of CTec3 and HTec3 enzymes were 1.19 and 1.21, respectively. The sugars present in the enzyme solutions as storage stabilizers [64] were accounted for in the calculations. The enzymatic cocktail was also supplemented with multifect pectinase (MP) (72 mg protein/mL, Batch No. 4861295753), a gift from Genencor (Pala Alto, CA, USA), which at this moment is no longer available.

### Microbial fermentation

To perform separate hydrolysis and fermentation (SHF) experiments, enzyme hydrolysis of all four AFEX-pretreated agave biomass materials (without any washing step) was carried out at 20% total solids, in 2 L baffled flasks with a total reaction mass of 400 g, an enzyme dosage of 20 mg protein/g glucan of Cellic^®^ CTec3, and 5 mg protein/g glucan Cellic^®^ HTec3 for pretreated agave bagasse. The enzyme dosage used for AFEX-pretreated agave leaf matter was 20 mg total protein/g glucan, using the same enzyme ratio. Hydrolysis was carried out for 72 h at 50 °C and 250 rpm, and the pH was adjusted to 5.0 using 12.0 M hydrochloric acid.

The hydrolysate slurry was centrifuged in 500 mL bottles at 7500 rpm for 30 min and the initial supernatant pH was adjusted to 5.5 using 10 M potassium hydroxide and then sterile-filtered using a 0.22 μm Stericup (Millipore™, USA). Sterile-filtered hydrolysate was used as is for microbial fermentation without any detoxification, conditioning, or nutrient supplementation. Most fermentation experiments were carried out in 125 mL Erlenmeyer flasks using 50 mL of liquid hydrolysate and *S. cerevisiae* 424A (LNH-ST), a xylose-fermenting yeast strain obtained from Prof. Nancy W. Y. Ho, Purdue University, West Lafayette, IN, USA, that can co-ferment xylose into ethanol. The yeast was previously grown on YEP media before transferring to agave hydrolysate at an initial OD of 2.0 (~ 0.95 g/L). Cell densities were measured using a UV/Vis Spectrophotometer (Beckmann Coulter DU720) at a wavelength of 600 nm. Fermentation tests were carried out in a shaking incubator at 150 rpm, a temperature of 30 °C, and pH 5.5 for a period of 48–72 h. About 500 µL of slurry was drawn at regular intervals, diluted, and filter-conditioned for the measured sugar and ethanol concentrations using HPLC.

## Additional file

**Additional file 1:** **Figure S1.** Box and whisker plot for sugar yields at varying AFEX pretreatment conditions on Agave biomass. Here, % sugar yields (glucose and xylose) from the whole set of experiments (DoE) for each biomass, minimum and maximum values, as well as the interquartile range, are shown. Enzyme hydrolysis was carried at 1% glucan loading, using Cellic^®^ CTec2 (9 mg protein/g glucan) and Cellic^®^ HTec2 (6 mg protein/g glucan), pH 4.8, 250 rpm and 50 °C. **Figure S2.** SEM Images for untreated and AFEX-pretreated *A. tequilana* bagasse. Here, untreated (left), AFEX treated (right). **Figure S3.** Effects of AFEX parameters on monomeric sugar conversions from *A. salmiana* bagasse. Here, glucan conversion A to C (green) and xylan conversion D to F (blue). **Figure S4.** Effects of AFEX parameters on monomeric sugar conversions from fibers of two agave species leaves. Here, glucan conversion A to C (green) and xylan conversion D to F (blue). **Figure S5.** Ternary contour plots showing effects of varying the ratio of commercial enzymes on sugar conversion. Glucan conversion (left column) and xylan conversion (right column) to monomeric sugars from the four AFEX-pretreated agave feedstocks. Here, (a) *A. tequilana* bagasse, (b) *A. tequilana* leaf fibers, (c) *A. salmiana* bagasse and (d) *A. salmiana* leaf fibers. Enzymatic hydrolysis was conducted at different enzyme mixtures with CTec3, HTec3 and Multifect Pectinase at 6% glucan loading, at total enzyme loading of 20 mg of protein/g glucan, pH 5.0, 250 rpm and 72 h. **Figure S6.** Monomeric glucose release during high solids loading EH of pretreated *A. tequilana* leaf fiber. As a function of enzyme loading at 20% total solids. **Figure S7.** Different untreated and pretreated agave residues. Here, *A. tequilana* bagasse (untreated) (A), *A. tequilana* bagasse (AFEX treated) (B), A. salmiana bagasse (untreated) (C), *A. salmiana* bagasse (AFEX treated) (D), *A. tequilana* leaf fibers (untreated) (E), *A. tequilana* leaf fibers (AFEX treated) (F), *A. salmiana* leaf fibers (untreated) (G) and *A. salmiana* leaf fibers (AFEX treated) (H). **Table S1.** AFEX conditions tested in the statistical design of experiments performed on each agave biomass. **Table S2.** Regression Coefficients of Mixture Design Model from the Enzyme ratio optimization of pretreated Agave residues.

## Abbreviations

AFEX: ammonia fiber expansion
DoE: design of experiments
HMF: 5-hydroxymethyl-furfural
LCC: lignin–carbohydrate complex
ASB: *Agave salmiana* bagasse
ASL: *Agave salmiana* leaf (lignocellulosic)
ATB: *Agave tequilana* bagasse
ATL: *Agave tequilana* leaf (lignocellulosic)
SHF: separate hydrolysis and fermentation
WSC: water-soluble carbohydrates
YEP: yeast extract phosphate
HPLC: high-performance liquid chromatography
RSM: response surface methodology
GHG: greenhouse gas

## Footnotes

1 TM—AFEX is a trademark of MBI International, Lansing, Michigan.
