# Supplementary material for: Conversion of lignocellulosic agave residues into liquid biofuels using an AFEX™-based biorefinery
Source: Biotechnol Biofuels. 2018 Jan 16;11:7. doi: 10.1186/s13068-017-0995-6 (PMC5769373; doi:10.1186/s13068-017-0995-6)
Supplement: Supplementary file 1 — Additional file 1: Figure S1. Box and whisker plot for sugar yields at varying AFEX pretreatment conditions on Agave biomass. Here, % sugar yields (glucose and xylose) from the whole set of experiments (DoE) for each biomass, minimum and maximum values, as well as the interquartile range, are shown. Enzyme hydrolysis was carried at 1% glucan loading, using Cellic® CTec2 (9 mg protein/g glucan) and Cellic® HTec2 (6 mg protein/g glucan), pH 4.8, 250 rpm and 50 °C. Figure S2. SEM Images for untreated and AFEX-pretreated A. tequilana bagasse. Here, untreated (left), AFEX treated (right). Figure S3. Effects of AFEX parameters on monomeric sugar conversions from A. salmiana bagasse. Here, glucan conversion A to C (green) and xylan conversion D to F (blue). Figure S4. Effects of AFEX parameters on monomeric sugar conversions from fibers of two agave species leaves. Here, glucan conversion A to C (green) and xylan conversion D to F (blue). Figure S5. Ternary contour plots showing effects of varying the ratio of commercial enzymes on sugar conversion. Glucan conversion (left column) and xylan conversion (right column) to monomeric sugars from the four AFEX-pretreated agave feedstocks. Here, (a) A. tequilana bagasse, (b) A. tequilana leaf fibers, (c) A. salmiana bagasse and (d) A. salmiana leaf fibers. Enzymatic hydrolysis was conducted at different enzyme mixtures with CTec3, HTec3 and Multifect Pectinase at 6% glucan loading, at total enzyme loading of 20 mg of protein/g glucan, pH 5.0, 250 rpm and 72 h. Figure S6. Monomeric glucose release during high solids loading EH of pretreated A. tequilana leaf fiber. As a function of enzyme loading at 20% total solids. Figure S7. Different untreated and pretreated agave residues. Here, A. tequilana bagasse (untreated) (A), A. tequilana bagasse (AFEX treated) (B), A. salmiana bagasse (untreated) (C), A. salmiana bagasse (AFEX treated) (D), A. tequilana leaf fibers (untreated) (E), A. tequilana leaf fibers (AFEX treated) (F), A. salmi [file 13068_2017_995_MOESM1_ESM.docx]

**Conversion of Lignocellulosic Agave Residues into Liquid Biofuels using AFEX**

Flores-Gómez Et al. (2017).

**Additional Figures and Tables**


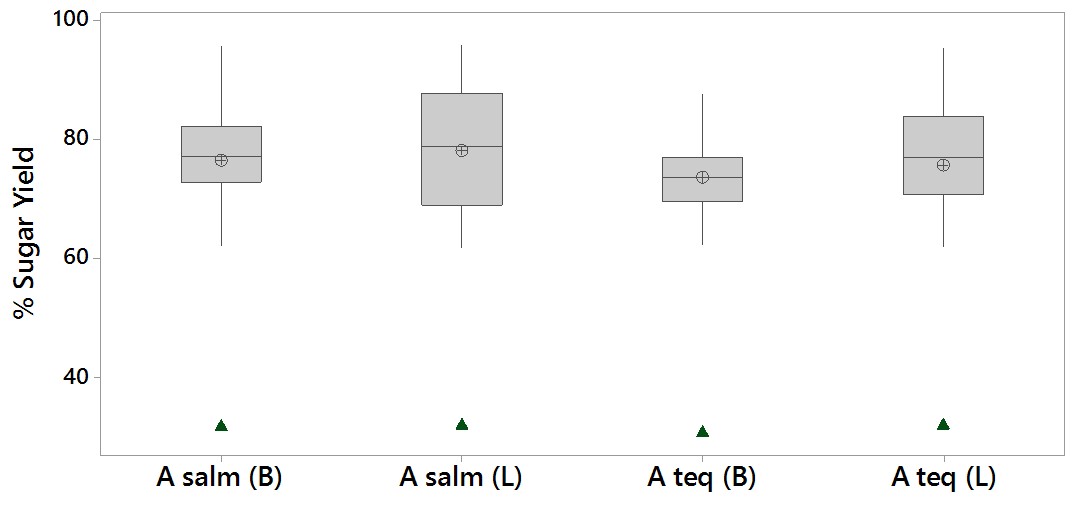


**Figure S1.** Box and whisker plot for sugar yields at varying AFEX pretreatment conditions on Agave biomass. Here, % sugar yields (glucose and xylose) from the whole set of experiments (DoE) for each biomass, minimum and maximum values, as well as the interquartile range, are shown. Enzyme hydrolysis was carried at 1% glucan loading, using Cellic^®^ CTec2 (9 mg protein/g glucan) and Cellic^®^ HTec2 (6 mg protein/g glucan), pH 4.8, 250 rpm and 50°C.


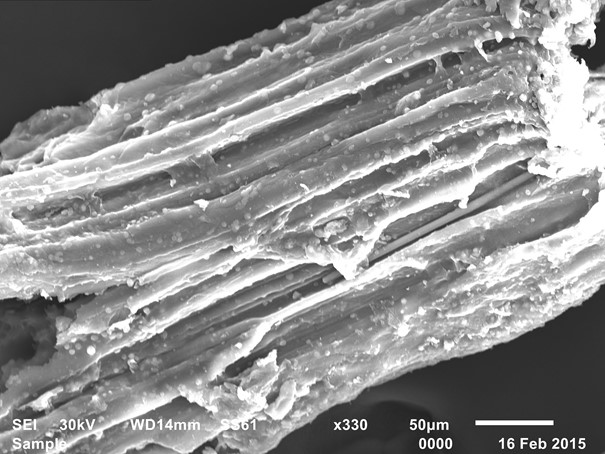

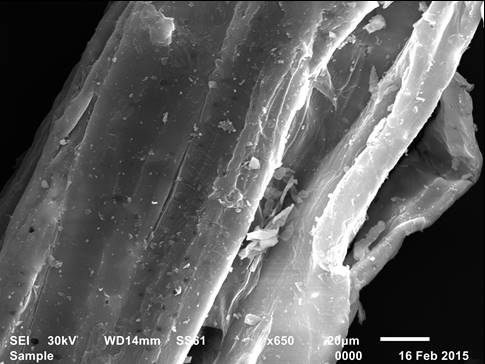


**Figure S2.** SEM Images for untreated and AFEX-pretreated *A. tequilana* Bagasse. Here, Untreated (left), AFEX treated (right).


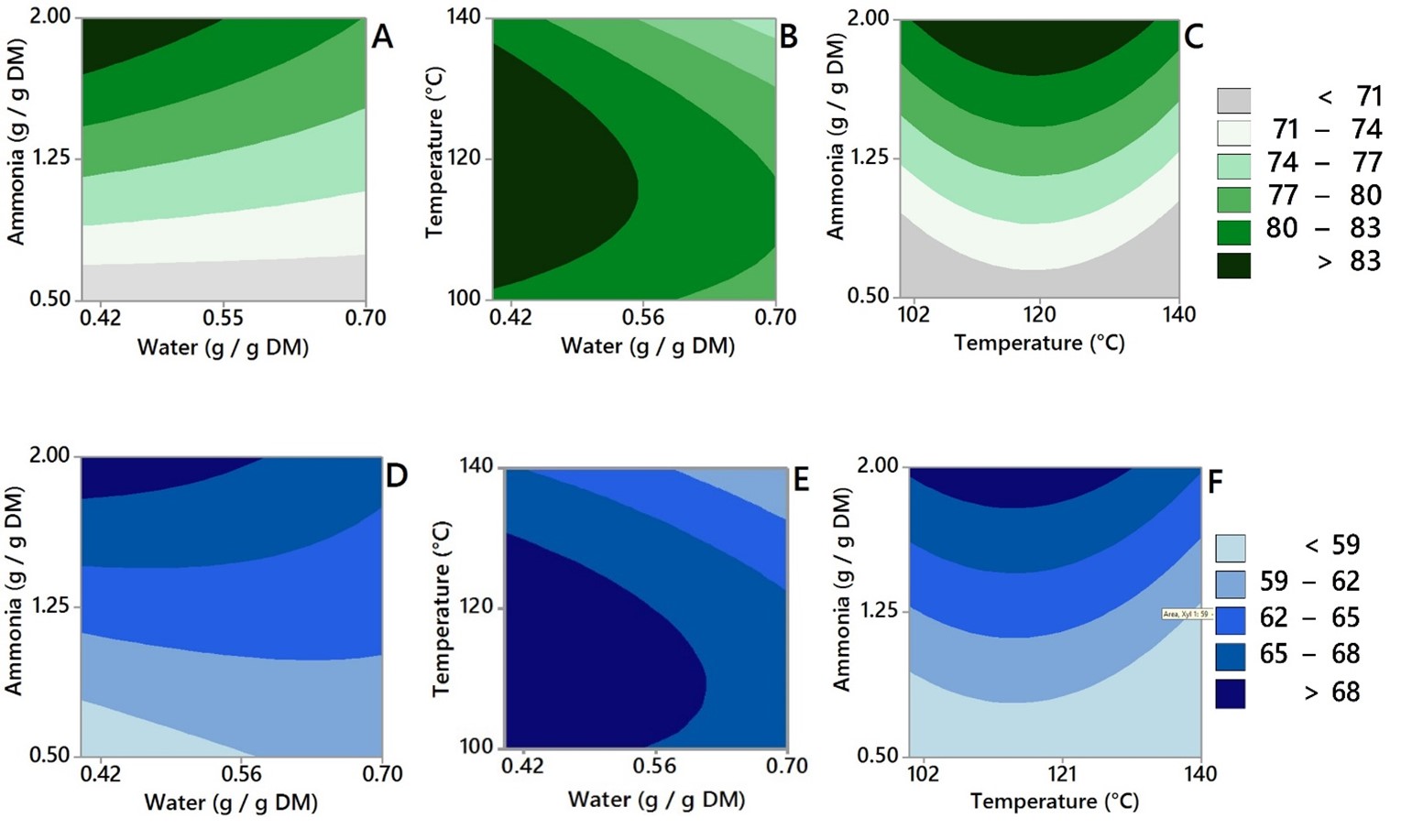


**Figure S3.** Effects of AFEX parameters on monomeric sugar conversions from *A. salmiana* bagasse. Here, glucan conversion A to C (green) and xylan conversion D to F (blue).

**
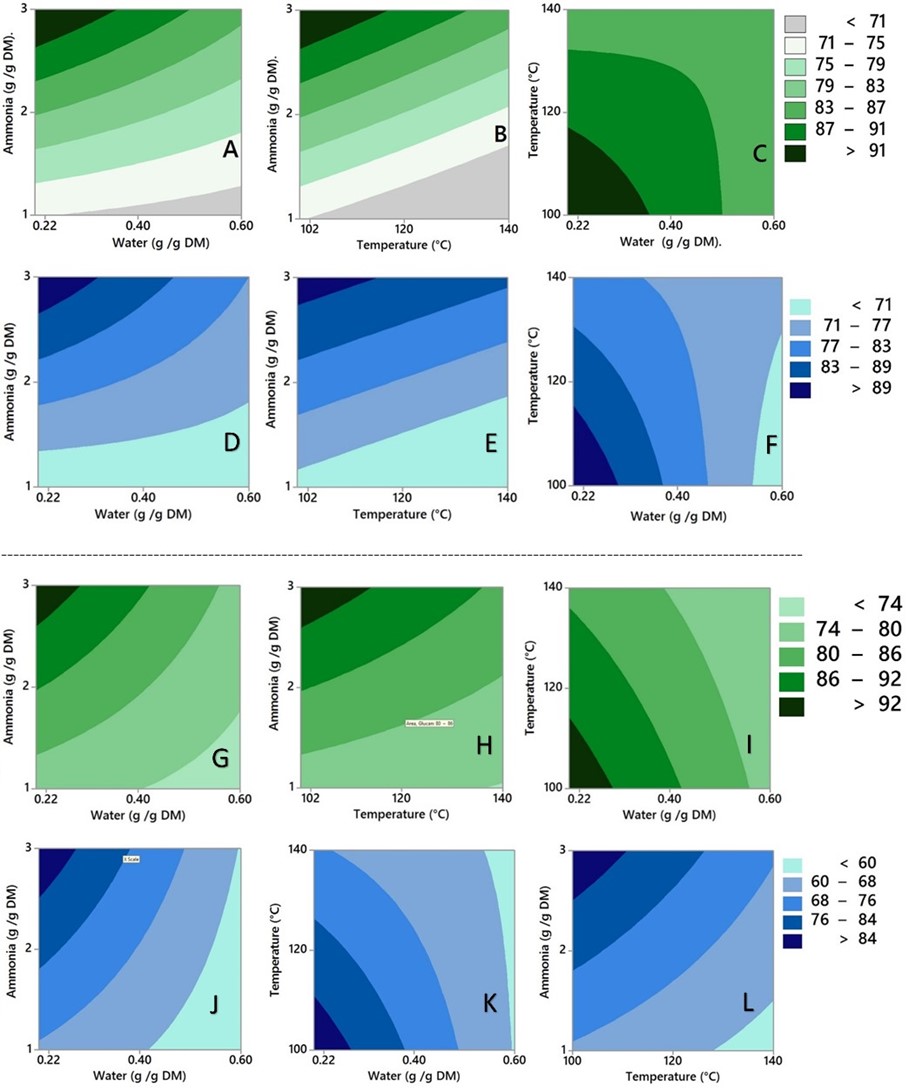
**

**Figure S4.** Effects of AFEX parameters on monomeric sugar conversions from fibers of *A. tequilana* leaf (A to F), and *A. salmiana* leaf (G to L). Glucan conversion (green): A to C and G to I; and xylan conversion (blue): D to F and J to L


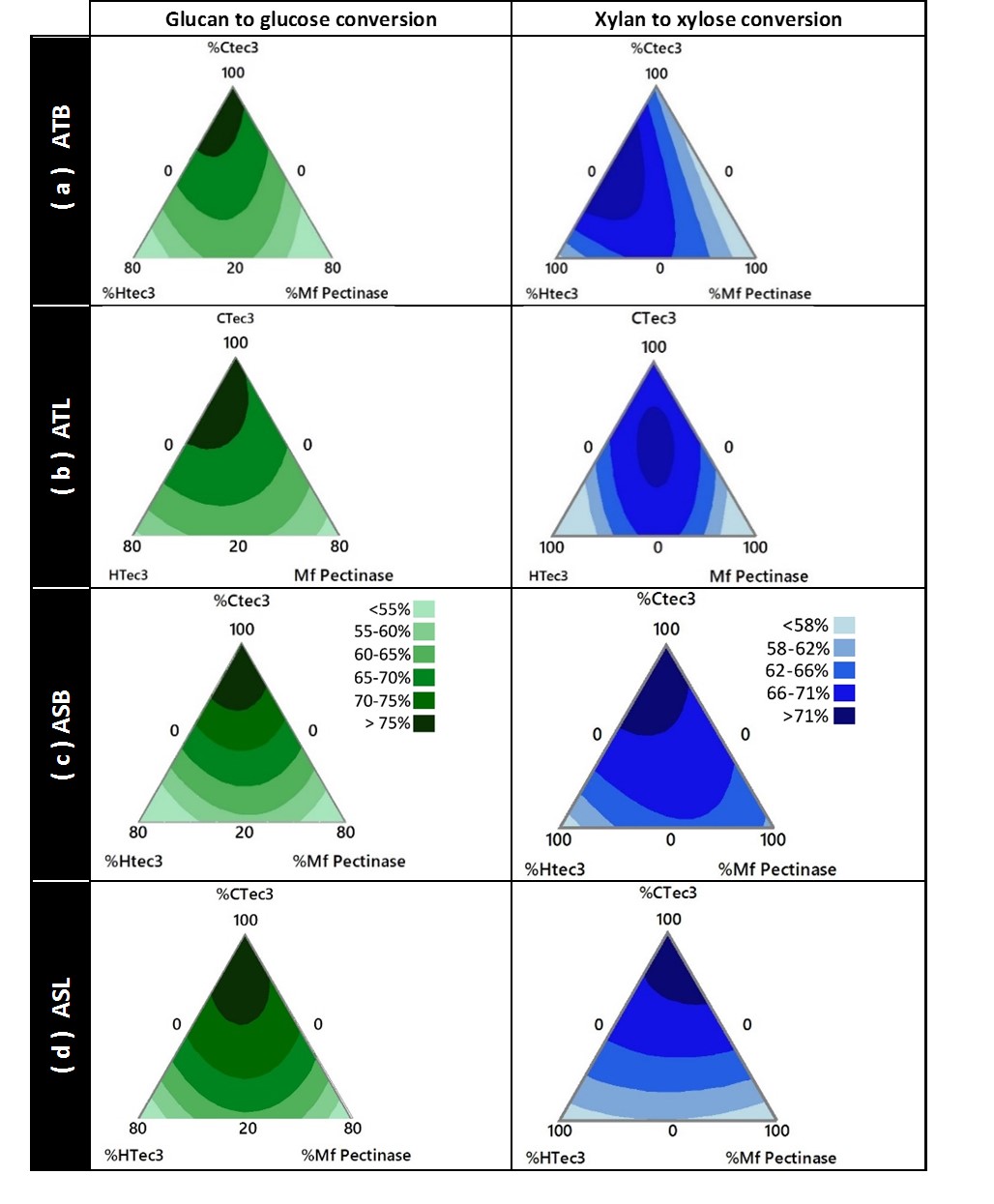


**Figure S5.**  Ternary contour plots showing effects of varying the ratio of commercial enzymes on sugar conversion. Here, Glucan conversion (left column) and xylan conversion (right column) to monomeric sugars from the four AFEX-pretreated agave feedstocks: (a) *A. tequilana* bagasse, (b) *A. tequilana* leaf fibers, (c) *A. salmiana* bagasse and (d) A. *salmiana* leaf fibers. Enzymatic hydrolysis was conducted at different enzyme mixtures with CTec3, HTec3 and Multifect-pectinase at 6% glucan loading, at total enzyme loading of 20 mg of protein/g glucan, pH 5.0, 250 rpm and 72 h.


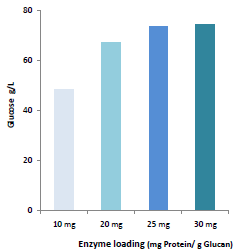


**Figure S6** Monomeric glucose release during high solids loading EH of pretreated *A. tequilana* leaf fiber.

As a function of enzyme loading at 20 % total solids.


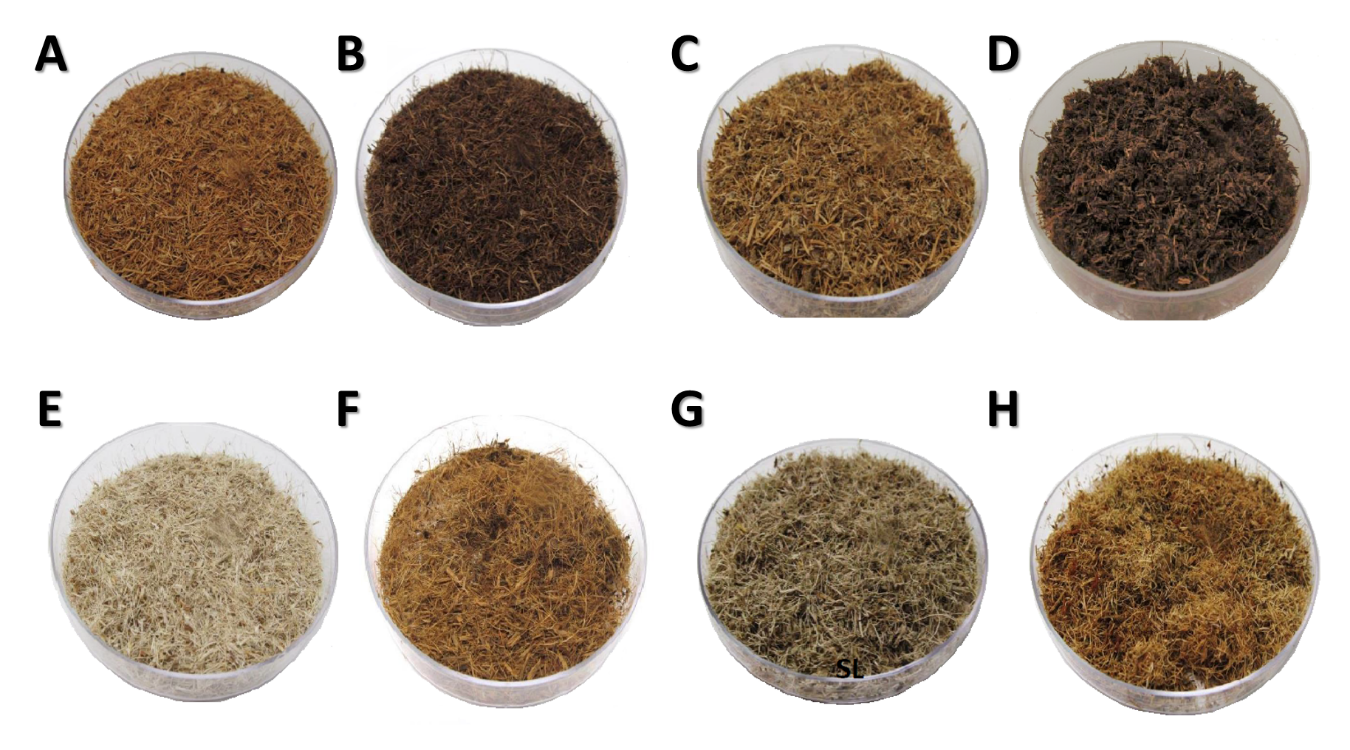


**Figure S7** Different untreated and pretreated agave residues. Here, *A. tequilana* bagasse (untreated) (A), *A. tequilana* bagasse (AFEX treated) (B), *A. salmiana* bagasse (untreated) (C), *A. salmiana* bagasse (AFEX treated) (D), *A. tequilana* leaf fibers (untreated) (E), *A. tequilana* leaf fibers (AFEX treated) (F), *A. salmiana* leaf fibers (untreated) (G) and *A. salmiana* leaf fibers (AFEX treated) (H).

**Table S1.** AFEX conditions tested in the statistical design of experiments performed on each agave biomass.

| Feedstock | |  | Bagasse ^a,b^ | | |  | Leaf fibers ^c^ | |
| --- | --- | --- | --- | --- | --- | --- | --- | --- |
| **Factor** | |  | **-1** | **0** | **+1** |  | **-1** | **+1** |
| Ammonia | (g NH_3_ /g DM) |  | 0.5 | 1.55 | 2 |  | 1 | 3 |
| Temperature | (°C) |  | 100 | 120 | 140 |  | 100 | 140 |
| Moisture | (g H_2_O / g DM) |  | 0.4 | 0.55 | 0.7 |  | 0.2 | 0.6 |
| Residence time^§^ | (min) |  | 16 | 38 | 60 |  | 30 | 30 |

Levels: (-1) Low, (0) Center and (+1) High conditions.

1. A. tequilana bagasse: 4 Factors Box-Behnken DoE.
2. A. salmiana bagasse: 3 Factors Box-Behnken DoE. Residence time held constant at 30 min.
3. Leaf fibers for the two agave species were tested with 2^3^ Full factorial DoE. Residence time held constant at 30 min.

**Table S2.** Regression Coefficients of Mixture Design Model from the Enzyme ratio optimization of pretreated Agave residues.

|  | *A. tequilana* bagasse | |  | *A. salmiana* bagasse | |  | *A. tequilana*  leaf fiber | |  | *A. salmiana* leaf fiber | |
| --- | --- | --- | --- | --- | --- | --- | --- | --- | --- | --- | --- |
| **Term** | **Coef** | **p** |  | **Coef** | **p** |  | **Coef** | **p** |  | **Coef** | **p** |
| **β_1_** | 66.59 | * |  | 76.38 | * |  | 73.69 | * |  | 72.53 | * |
| **β_2_** | 15.03 | * |  | 18.94 | * |  | 12.72 | * |  | 15.70 | * |
| **β_3_** | 16.94 | * |  | 28.55 | * |  | 15.31 | * |  | 18.41 | * |
| **β_12_** | 82.85 | 0.000 |  | 60.42 | 0.000 |  | 96.4 | 0.000 |  | 60.48 | 0.000 |
| **β_13_** | 46.93 | 0.000 |  | 45.27 | 0.000 |  | 52.63 | 0.000 |  | 67.46 | 0.000 |
| **β_23_** | 65.9 | 0.020 |  | 99.85 | 0.002 |  | 110.57 | 0.001 |  | 122.5 | 0.000 |

**1** = CTec3^a^, **2** = HTec3^a^ and **3**= Multifect^-^Pectinase^b^.

1. Novozymes, Denmark, (b) Genencor-Dupont, US.
